# Three-Dimensional Preoperative Planning of Corrective Osteotomies for Distal Radius Malunions: A Systematic Review of Clinical and Radiographic Outcomes

**DOI:** 10.1177/15589447251352001

**Published:** 2025-08-12

**Authors:** Khaled Skaik, Abdulrahman Khizindar, Qasim Mughal, Marie Gdalevitch, Jennifer Mutch

**Affiliations:** 1 Faculty of Medicine and Health Sciences, McGill University, Montreal, QC, Canada; 2 Division of Orthopaedic Surgery, McGill University, Montreal, QC, Canada; 3 Verdun Hospital, Montreal, QC, Canada; 4 Saint Mary’s Hospital Center, Montreal, QC, Canada

**Keywords:** wrist, post traumatic, distal radius, malunion, fracture/dislocation, outcomes, treatment, surgery

## Abstract

Distal radius malunions (DRMs) are painful and functionally impairing, often necessitating surgical intervention to restore wrist anatomy and function. Traditional corrective osteotomies, which rely on orthogonal radiographs, may overlook complex deformities. This study aims to evaluate the techniques and effectiveness of 3-dimensional (3D)-planned corrective osteotomies, along with their clinical and radiographic outcomes. We conducted a systematic review of literature across PubMed, Ovid, EMBASE, and Web of Science for studies that implemented 3D planning in corrective osteotomies for DRM. We identified 792 articles, of which 24 met the inclusion criteria with a total of 199 corrective osteotomies of symptomatic DRM, of which 127 (64%) were extra-articular and 39 (19.5%) intra-articular, with the remaining being a combination of intra-articular and extra-articular or unspecified. To transfer 3D preoperative plan to patient, 18 out of 24 used 3D-printed patient-specific cutting guides for intraoperative guidance. Two studies implemented the transfer of the preoperative plan using simulated osteotomies on 3D-printed models, while one study used a dynamic referencing body to match real-time surgical actions with the virtual plan. The majority (98.5%, n = 196) demonstrated statistical significance in achieving the acceptable limits of radial inclination (21°-25°), ulnar variance (<3 mm), and volar tilt (≤15° dorsal and ≤20° volar). Functional outcomes significantly improved in all studies (*P* < .05). Complications were reported in 22 cases (11%) and included partial laceration of the extensor pollicis longus tendon, hardware problems requiring removal, and screw loosening. Future research should focus on balancing the technique’s additional costs and logistical demands with its potential long-term benefits.

## Introduction

Post-traumatic distal radius malunions (DRMs) can result in significant disability with patients presenting with pain, weakness, or functional limitations in the hand and wrist.^1^ While there is no precise correlation between radiographic abnormalities and clinical functional outcomes, surgical correction of DRM is a reasonable option when the degree and direction of radiographic displacement can explain the patient’s symptoms (eg, significant radial shortening resulting in ulnar-sided pain and disability).^1^ Achieving precise anatomical correction of a DRM remains a significant challenge but is recommended to optimize functional outcomes.^2^

Corrective osteotomy of the distal radius is the treatment of choice to restore anatomy and optimize functional outcome in DRM.^2^ The distal radius deformity can be addressed in all 3 spatial planes: palmar tilt in the sagittal plane, a positive radio-ulnar index in the vertical plane, and horizontal alignment (radial tilt) of the bi-styloid line in the frontal plane.^3^ However, preoperative planning to determine the necessary corrections and fixation of a complex 3-dimensional (3D) deformity remains challenging.^3^ Particularly, this planning has traditionally relied on 2-dimensional (2D) orthogonal radiographs (lateral and posteroanterior views of the distal radius), and this method might fall short in addressing the more complex elements of the deformities in DRM, such as rotation and translation, underscoring the need for advanced planning techniques.^4^

Computer-assisted techniques with 3-dimensional (3D) images and models address 3D deformity and may further optimize functional and radiographic results of corrective osteotomies.^5,6^ This usually involves 3 steps: (1) computed tomography (CT) scans of both the malunited and the healthy contralateral forearms are performed; (2) virtual models of both radii are generated and the malunited radius is overlaid onto a mirrored model of the healthy side to identify the location and extent of the deformity; and (3) a virtual cutting plane is then established within the malunited area and the proximal and distal segments are manipulated (rotation, angulation, translation) until they align with the contralateral radius.^5,6^ The planned osteotomy is then transferred and executed intraoperatively. This is a delicate process for which several solutions have been proposed.

One common approach is creating virtual or physical 3D models preoperatively that can help surgeons visualize and practice the planned osteotomy before the operative procedure.^3,7^

Another widely used approach is the creation of patient-specific cutting guides. These guides, designed for cutting or drilling, have been successfully implemented in previous studies^3,8-13^ and have been shown to be effective in accurately positioning surgical cuts and implants. These 2 methods—physical/virtual 3D models and patient-specific guides—are the most commonly employed techniques for translating preoperative plans into the operating room (OR).^5,8,10,11,13,14^ Advancements in computer technology and 3D printing have made 3D-guided osteotomy planning more accessible and more common in modern clinical practice and several methods or “recipes” have been proposed.

The aim of this study was to assess and summarize the results of corrective osteotomies of DRM with the use of 3D planning techniques by systematically evaluating the available literature. The objectives include to: (1) examine and describe the methodologies used in 3D-guided preoperative planning and intraoperative execution; (2) summarize the preoperative and postoperative clinical outcomes; (3) evaluate the radiological outcomes to accepted limits of deformity^15-17^; and (4) summarize the complications associated with these 3D-planned corrective osteotomies.

## Methods

This review was registered with the International Prospective Register of Systematic Reviews (PROSPERO, CRD42024587132) before starting. The Cochrane Handbook for Systematic Reviews of Interventions: Preferred Reporting Items for Systematic Reviews and Meta-analyses (PRISMA) guidelines were applied to assess the quality of the results published in all included studies to make sure the results of our systematic review were reliable.^18^

### Search Strategy

The search aimed to identify articles that focused on 3D-guided virtual planning of corrective osteotomies of the distal radius. A comprehensive search was performed across multiple electronic databases, including Web of Science, Embase, PubMed, and Ovid Medline covering studies up to 29 October 2024. Keywords such as “Colles’ fracture,” “3-dimensional,” “corrective osteotomies,” and radius “malunion” were combined using Boolean operators “AND” and “OR.” Broader MeSH terms, such as “bones of the upper extremity,” were used to ensure comprehensive coverage and avoid missing any articles that discussed corrective osteotomies of the distal radius alongside osteotomies of other bones within the same study. The search strategies are seen in Supplemental Material. Screening and full-text reviews were performed using Covidence review management software.

### Inclusion Criteria

The inclusion criteria for the study were as follows:

- Studies describing patients with post traumatic DRM;
- Osteotomies described as “3D-planned” (preoperative planning based on computer-assisted 3D imaging of both the malunited and uninjured distal radius);
- Papers with preoperative and postoperative clinical or radiographic data or both.

Exclusion criteria were as follows:

- Deformities caused by growth disturbances or congenital anomalies (eg, Madelungs);
- Studies using 3D-planned corrective osteotomies exclusively on phantoms or cadavers;
- Studies or patients without at least one radiographic or clinical functional outcome reported preoperatively and postoperatively;
- Descriptive technical reports that did not perform any 3D-planned corrective osteotomies;
- Non-English articles;
- Unpublished studies;
- Articles focusing on reviews, commentaries, or editorials.

### Data Extraction

Two authors (KS and AK) independently screened the studies for inclusion and disagreements were resolved by discussion or by consulting with a third author (JM) as needed. The following data were then extracted: first author, year of publication, study design, number of participants, number of osteotomies, mean age, mean follow-up time, method of preoperatively planning the osteotomy, preoperative and postoperative clinical outcomes (range of motion [ROM] measurements [flexion/extension and pronation/supination], grip strength, Disabilities of the Arm, Shoulder, and Hand [DASH] score), postoperative complications, and preoperative and postoperative radiographic outcomes (ulnar variance, volar tilt, and radial inclination).

### Quality Assessment

The Joanna Briggs Institute (JBI) Critical Appraisal Checklists for case reports and case series was used to evaluate the quality of the included articles. The JBI checklist for case reports is an 8-item tool that assesses key elements such as the patient’s demographic information, medical history, current clinical condition, diagnostic tests, treatment details, post-intervention outcomes, adverse events, and key takeaways.^19^ The JBI checklist for case series consists of 10 items, focusing on inclusion criteria, methods for measuring the condition, diagnostic validity, consecutive participant inclusion, completeness of participant data, reporting of demographic and clinical information, outcomes, and the appropriateness of statistical analyses.^20^

## Results

### Included Studies

**Figure 1.**
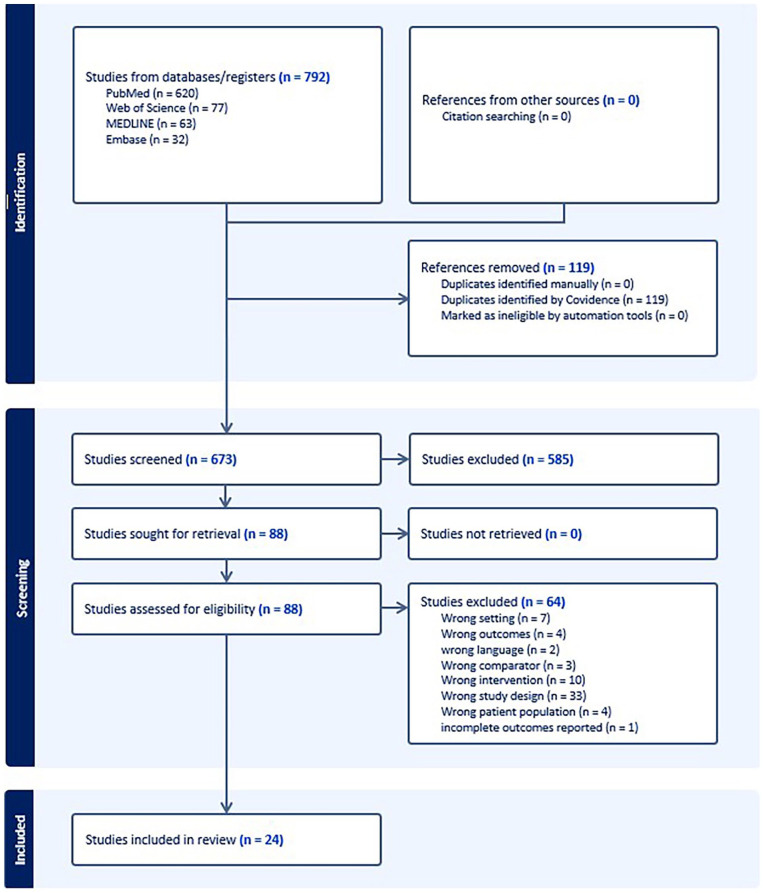
Preferred Reporting Items for Systematic Reviews and Meta-analyses flow diagram.

A total of 792 studies were identified by the reviewers (KS and AK). Of these studies, 119 studies were duplicates and 585 were excluded during the abstract review. Eighty-eight (88) studies underwent full-text review, and 24 final studies were retained for this systematic review (15 retrospective studies, 4 prospective case series, 5 case reports). A flowchart of the studies is provided in Figure 1.

### Quality Assessment

Among the case series, 2 studies achieved a perfect JBI score of 10/10, 3 studies had the lowest score of 7/10, and the overall mean score was 8.36/10. Most of the case series were considered to be of high quality, and the quality assessments are summarized in Supplemental Table S1.

Among the case reports, 1 out of 5 reports received a perfect JBI score of 8/8, while the remaining 4 scored 7/8, resulting in an overall mean score of 7.2/8. The quality assessments are summarized in Supplemental Table S2.

### Patient Population

The total number of patients included in this study was 199 patients with 199 corrective osteotomies. The largest proportion of patients were from Japan (32.7%, n = 65), followed by Switzerland (17.9%, n = 35). The remaining patients were from the Netherlands, Canada, Brazil, France, Austria, Belgium, and Norway. The mean age of participants was 42.8 years old (range, 12-79) at the time of surgery, with a mean postoperative follow-up of 18 months (range, 2-120 months). For 64% (n = 127) of participants, the DRM were extra-articular while 19.5% (n = 39) were intra-articular, 2% (n = 4) were combined intra-articular and extra-articular, and the remaining 29 DRM were not specified. This is summarized in Table 1.

**Table 1.** Characteristics of the Included Studies and Patient Population.

Study (reference)	Publication year	Country	Study design	Patients in study (N)	Number of osteotomies (N)	Type of fracture	Mean age (years)	Gender (% males)	Mean follow-up time
Haandrikman et al^8^	2024	The Netherlands	Retrospective case series	2	2	1 extra-articular and 1 intra-articular	33.3	33.3	47.3 weeks
Murase et al^7^	2008	Japan	Case report	1	1	Extra-articular	12	100	15 weeks
Bilic and Zdravkovic^21^	1994	Croatia	Retrospective case series	27	27	Extra-articular	NR	59.2	NR
Belloti et al^3^	2021	Brazil	Prospective case series	9	9	Intra-articular	47.5	55.5	10 months
Athwal et al^6^	2003	Canada	Retrospective case series	6	6	Not specified	50	66.7	25 months
Dobbe et al^22^	2021	The Netherlands	Prospective case series	10	10	Not specified	37	40	6 months
Singh et al^23^	2022	Switzerland	Retrospective case series	15	15	Intra-articular	49.2	46.7	6 years
Schindele et al^24^	2024	Switzerland	Retrospective case series	13	13	Extra-articular	56	30.8	1 year
Oka et al^25^	2019	Japan	Prospective, multicenter study	8	8	Extra-articular	40.4	12.5	52 weeks
Vlachopoulos et al^10^	2015	Switzerland	Retrospective cohort study	8	8	Extra-articular	23.2	37.5	12 months
Oka et al^26^ (volar)	2018	Japan	Retrospective comparative study	9	9	Extra-articular	55.8	11.1	>12 months
Oka et al^26^ (dorsal)	2018	Japan	Retrospective comparative study	19	19	Extra-articular	55.5	15.8	>12 months
Yoshii et al^27^	2021	Japan	Retrospective comparative study	10	10	Not specified	59.4	40	NR
Athlani et al^11^	2020	France	Prospective case series	16	16	Extra-articular	45	43.8	12 months
Honigmann et al^28^	2016	Switzerland	Case report	1	1	Intra-articular	54	100	6 weeks
Kunz et al^12^	2013	Canada	Case report	1	1	Extra-articular	61	0	6 weeks
Miyake et al^29^	2011	Japan	Retrospective case series	10	10	8 Extra-articular and 2 Intra-articular	56	0	16 months^a^
Rieger et al^30^	2005	Austria	Retrospective case series	11	11	Extra-articular	NR	NR	5.3 months
Schweizer et al^31^	2013	Switzerland	Retrospective case series	6	6	Intra-articular	48	66.7	1 year
Stockmans et al^32^	2013	Belgium	Retrospective case series	4	4	Combined intra-articular and extra-articular	54	25	NR
Walenkamp et al^33^	2015	The Netherlands	Retrospective case series	3	3	Extra-articular	45	0	32 months
Oka et al^34^	2008	Japan	Case report	1	1	Intra-articular	32	0	3 years
Oka et al^35^	2020	Japan	Retrospective case series	5	5	Intra-articular	44.4	100	48.5 months
Temmesfeld et al^13^	2020	Norway	Case report	1	1	Intra-articular	18	100	1 year
Oka et al^9^	2010	Japan	Retrospective case series	2	2	Extra-articular	33	50	26.5 months

*Note.* NR = not reported.

a Reported as median follow-up time.

### Preoperative 3D-Guided Planning

In all studies, a 3D construct from CT scans was made to plan the corrective osteotomies. In 2 studies that focused on intra-articular malunion, there was no mention of using the contralateral arm as a reference. In all other studies, bilateral CT scans were taken and the healthy and malunited arms were superimposed to plan the site and dimensions of the osteotomy.^13,34^ In all studies, osteotomies were planned preoperatively using specialized software that allowed for the simulation of rotational, opening, or closing wedge osteotomies. Most of the corrective osteotomies performed were opening wedge osteotomies (n = 115), followed by closed wedge (n = 13) and translational osteotomies (n = 8). The type of corrective osteotomy was not clear in the remaining patients.

### Transfer of Preoperative Plan to Patient

Most of the studies (18 out of 24) used 3D-printed patient-specific cutting guides for performing osteotomies.^8-13,22-26,28,29,31-35^ Two studies executed the osteotomy on a 3D-printed model of the patient’s malunion before replicating the same procedure on the actual patient.^3,7^ More specifically, Murase et al^7^ performed a simulated osteotomy on a patient-specific 3D-printed stereolithography model with an external fixator, aligning it according to the calculated axis of deformity from computer simulation, before applying the procedure to the patient. Athwal et al^6^ employed a dynamic referencing body (of infrared-emitting diodes [IREDs]) to track the real-time position of the patient’s bone, aligning it with the preoperative 3D model to precisely guide osteotomy cuts and implant positioning. Similarly, Rieger et al^30^ used a 3D-printed model equipped with a repositioning device tailored to match the planned osteotomy. This repositioning device, when placed in the osteotomy gap during the surgical operation, would restore the original position of the distal articular radius fragment. This device was subsequently removed and replaced with a bone graft to fill the osteotomy gap.^30^

Bilic and Zdravkovic^21^ and Oka et al^9^ prepared bone grafts using virtual simulation measurements, performing the osteotomy manually. In contrast, Honigmann et al^28^ also used customized bone grafts, but these were prepared with the assistance of patient-specific surgical guides. One study did not transfer their 3D-guided preoperative plan to a physical model like the rest of the studies.^27^ Instead, they had 2 groups of patients: one group transferred the 3D preoperative plan by overlaying digitally reconstructed radiographs onto live fluoroscopic images for real-time guidance during surgery; the other group relied on manually comparing static 3D preoperative plan images with fluoroscopy without real-time integration.^27^ Regarding the fixation method, most studies favored using standard implants with either volar or dorsal plating. This is summarized in Table 2.

**Table 2.** Transfer of the 3D-Guided Preoperatively Planned Osteotomy to the Operating Room.

Authors (reference)	Intraoperative transfer of preoperative plan
Haandrikman et al^8^	Patient-specific cutting guides
Murase et al^7^	A simulated osteotomy on a patient-specific 3D-printed model with an external fixator before replicating the procedure to the patient
Bilic and Zdravkovic^21^	Measurements from virtual osteotomy were used to create customized bone grafts, which were used to guide intraoperative osteotomy
Belloti et al^3^	A simulated osteotomy on a patient-specific 3D-printed model then replicated on the patient
Athwal et al^6^	An intraoperative guidance system that linked real-time surgical tool positions to the preplanned 3D model
Dobbe et al^22^	Patient-specific cutting guides
Singh et al^23^	Patient-specific cutting guides
Schindele et al^24^	Patient-specific cutting guides
Oka et al^25^	Patient-specific cutting guides
Vlachopoulos et al^10^	Patient-specific cutting guides
Oka et al^26^	Patient-specific cutting guides
Yoshii et al^27^	Group 1: Overlaying preoperatively planned reconstructed radiographs onto live fluoroscopic images. Group 2: Manually comparing static preoperatively planned images to fluoroscopy
Athlani et al^11^	Patient-specific cutting guides
Honigmann et al^28^	Patient-specific cutting guides
Kunz et al^12^	Patient-specific cutting guides
Miyake et al^29^	Patient-specific cutting guides
Rieger et al^30^	3D-printed repositioning device tailored to match the 3D-planned osteotomy
Schweizer et al^31^	Patient-specific cutting guides
Stockmans et al^32^	Patient-specific cutting guides
Walenkamp et al^33^	Patient-specific cutting guides
Oka et al^34^	Patient-specific cutting guides
Oka et al^35^	Patient-specific cutting guides
Temmesfeld et al^13^	Patient-specific cutting guides
Oka et al^9^	Patient-specific cutting guides

*Note*. 3D = 3-dimensional.

### Clinical Functional Outcomes

All studies showed significant improvement in ROM measurements as well as grip strength and DASH scores after the corrective osteotomy. Eleven studies reported the individual ROM measurements for preoperative and postoperative flexion, extension, pronation, and supination separately, while 7 papers reported these measurements together as a sum. A total of 9 studies reported preoperative and postoperative grip strength, with four expressing it as a percentage of the strength of the contralateral arm, while the remaining studies presented it in kilograms for the injured arm both before and after surgery. Only a total of 3 studies reported DASH scores, which all showed improvement postoperatively.^3,11,13^ These results are summarized in Table 3.

**Table 3.** Clinical Functional Outcomes of the Included Studies.

Authors (reference)	N	ROM wrist		ROM forearm		Grip strength (kg)		DASH score		Complications
		Flexion/extension (°)		Pro-/supination (°)						
		Pre-op	Post-op	Pre-op	Post-op	Pre-op	Post-op	Pre-op	Post-op	
Haandrikman et al^8^	2	65/42.5	65/62.5	60/57.5	70/82.5	—	45.5	—	—	Wound dehiscence
Murase et al^7^	1	20/—	60/80	—/—	80/80	—	—	—	—	None
Bilic and Zdravkovic^21^	27	—/—	—/—	—/—	—/—	—	—	—	—	None
Belloti et al^3^	9	—/—	—/—	—/—	—/—	—	—	61.65	24.9	None
Athwal et al^6^	6	—/—	47/42	—/—	78%/74%	—	30	—	14	An iatrogenic partial laceration of the extensor pollicis longus tendon
Dobbe et al^22^	7	95^a^	100^a^	95^b^	150^b^	17.5^c^	25.4^c^	—	—	4 hardware removals after few months. Screws broke after few months in 2 separate cases
Singh et al^23^	14	41/47.3	52.3/53	65.71/68.21	75.71/76.43	—	—	—	10.29	Osseous spur removal
Schindele et al^24^	13	90^d^	130^d^	135^e^	160^e^	26^f^	28^f^	—	—	Wound dehiscence
Oka et al^25^	8	44.4/53.1	73.8/70.6	69.4/68.8	83.8/83.1	—	—	—	—	None
Vlachopoulos et al^10^	8	—/—	—/—	—/—	—/—	—	—	—	—	None
Oka et al^26^	9	82^a^	121^a^	128^b^	158^b^	38%	83%	—	—	1 residual pain, 1 extensor adhesion, 4 dorsal wrist discomfort
Oka et al^26^	19	98^a^	132^a^	136^b^	162^b^	51%	84%	—	—	2 wrist discomfort
Yoshii et al^27^	10	—/—	—/—	—/—	—/—	—	—	—	—	None
Athlani et al^11^	16	100^a^	145^a^	105^b^	155^b^	20	40	57	17	None
Honigmann et al^28^	1	70/40	70/70	70/40	70/80	—	—	—	—	None
Kunz et al^12^	1	—/—	—/—	—/—	—/—	—	—	—	—	1 deep infection
Miyake et al^29^	10	32.5/62.5	62.5/66.5	71/76	81/84	39%	82%	—	—	2 post-op screw loosening and 1 EPL tendon problem
Rieger et al^30^	11	63/59	76/75	50/53	63/65	—	—	—	—	None
Schweizer et al^31^	5	36.7/49.2	55.8/61.7	69/55	76/80	30.83	41	—	—	None
Stockmans et al^32^	4	—/—	—/—	—/—	—/—	—	—	—	—	None
Walenkamp et al^33^	3	153^a^	153^a^	165^b^	175^b^	—	97%	—	—	1 distal radioulnar subluxation persisted
Oka et al^34^	1	5/45	70/80	—/—	—/—	22	45	—	—	None
Oka et al^35^	5	93^a^	146^a^	144^b^	164^b^	54%	85.8%	—	—	None
Temmesfeld et al^13^	1	75/75	82/87	85/90	90/90	—	38.2	23	7	None
Oka et al^9^	2	83^a^	113^a^	120^b^	150^b^	—	—	—	—	1 implant removal

*Note.* % given as percentage of the contralateral side. ROM = range of motion; DASH = Disabilities of the Arm, Shoulder, and Hand; Pre-op = preoperative; Post-op = postoperative; — = not reported; EPL = extensor pollicis longus.

a Total flexion-extension mean.

b Total pronation-supination mean.

c n = 6 instead of n = 7.

d Flexion-extension arc of motion median.

e Pronation-supination arc of motion median.

f Grip strength median.

Notably, 4.5% of patients (n = 9) were pediatric (age < 18). Among them, 8 had extra-articular fractures without evidence of physeal arrest or growth disturbance, all demonstrating improved clinical outcomes.^10,22,24^ Only one patient, reported by Murase et al^7^ had an intra-articular fracture with partial physeal arrest, with wrist flexion improving from 20° preoperatively to 60° postoperatively.

### Radiographic Outcomes

The mean values of radial inclination, ulnar variance, and volar tilt, as measured from anteroposterior and lateral radiographic views, are presented in Table 4. All studies showed mean postoperative radiological values that were within the established acceptable limits of deformity. When looking at the individual data of the cases where available, only 3 patients did not achieve acceptable deformity correction.^3^ A total of 9 studies demonstrated improvements from the corrective osteotomy exclusively in their clinical outcome measures including improvements in postoperative ROM, grip strength, or DASH score, with no corresponding evidence of improvement in radiographic outcomes.^7,10,22-24,27,31,33,34^ All pediatric patients also showed improved outcomes in radiographic measures.^7,10,22,24^

**Table 4.** Mean Radiographic Outcomes of the Included Studies.

Study (reference)	N	Radial inclination (°)		Ulnar variance (mm)		Volar tilt (°)			
		Pre-op	Post-op	Pre-op	Post-op	Pre-op		Post-op	
						Dorsal	Volar	Dorsal	Volar
Haandrikman et al^8^	2	22	27	6	1	25.5	N/A	5.5	N/A
Bilic and Zdravkovic^21^	27	2.8	16.5	—	—	−17.1	N/A	6.8	N/A
Belloti et al^3^	9	13.22	15.22	—	—	−15.88		9.33	
Athwal et al^6^	2	12	21	7.5	1.9	N/A	21	N/A	9
Athwal et al^6^	4	—	—	—	—	−30	N/A	9	N/A
Oka et al^25^	8	—	—	2.81	0.2	—	—	—	—
Oka et al^26^	9	−12.2^a^	−1.0^a^	3.4^a^	−0.1^a^	−37.1^a^	N/A	−1.2^a^	N/A
Oka et al^26^	19	−10.9^a^	−1.5^a^	4.4^a^	0.4^a^	N/A	−37.7^a^	N/A	−9.4^a^
Athlani et al^11^	8	19	21	2	−0.3	21	N/A	−3.3	N/A
Athlani et al^11^	8	—	—	2	−0.3	N/A	−29	N/A	-9
Honigmann et al^28^	1	25	25	5	−1	N/A	35	N/A	9
Kunz et al^12^	1	22	26	5	−2	39	N/A	4	N/A
Miyake et al^29^	10	13.4	24	6	1.1	−27	N/A	13	N/A
Rieger et al^30^	11	19.6	21.5	5.9	0.5	−31	N/A	10.3	N/A
Rieger et al^30^	11	19.6	21.5	5.9	0.5	N/A	26	N/A	10.3
Stockmans et al^32^	4	—	21	—	−2	N/A	4	—	—
Oka et al^35^	5	5.4^a^	1.2^a^	2.4^a^	0.4^a^	7.0^a^		2.8^a^	
Temmesfeld et al^13^	1	25	28	35	50	—	—	—	—
Oka et al^9^	2	12^a^	1^a^	—	—	N/A	28^a^	N/A	—

*Note.* N/A = not applicable; Pre-op = preoperative; Post-op = postoperative; — = not reported.

a Data given as difference to the contralateral side.

### Intra-articular Fractures

A total of 10 studies described 39 patients with intra-articular DRMs and an additional 4 patients with combined intra-articular and extra-articular DRMs.^32^ Intra-articular step-off and gap data were available for 30 patients, all of whom demonstrated significant improvement in these measures. This is summarized in Table 5.

**Table 5.** Radiographic Results of Intra-articular Fractures.

Study (reference)	N	Intra-articular step-off (mm)		Intra-articular gap (mm)	
		Pre-op	Post-op	Pre-op	Post-op
Haandrikman et al^8^	1	—	—	—	—
Belloti et al^3^	9	—	—	—	—
Singh et al^23^	14	2.3	0.7	—	—
Honigmann et al^28^	1	—	—	—	—
Miyake et al^29^	2	—	—	—	—
Schweizer et al^31^	5	2.7	0.7	—	—
Stockmans et al^32^	4	2.1	1.3	2.6	2.1
Oka et al^34^	1	3.0	0	—	—
Oka et al^35^	5	4.9	1.0	—	—
Temmesfeld et al^13^	1	3.3	0	2	0

*Note.* Pre-op = preoperative; Post-op = postoperative; — = not reported.

### Complications

Ten out of the 24 studies (n = 22, 11% of patient population) reported complications post-surgery, which varied in severity. These included 1 iatrogenic partial laceration of the extensor pollicis tendon, 3 hardware removals, 3 broken or loose screws, 5 hardware removals due to discomfort, tendon damage, or as part of a secondary corrective surgery to address residual deformities.^6,22,29,30^ Other problems included residual pain, extensor adhesion, dorsal wrist discomfort, or persistent radioulnar subluxation.^12,26,29,33^ This is summarized in Table 3.

## Discussion

All studies demonstrated that a 3D-planned corrective osteotomy significantly improved both functional and radiographic outcomes in patients with DRM. Most authors noted that conventional 2D planning techniques often fail to address 3D deformities adequately and encouraged the use of 3D preoperative planning.

The 3D preoperative virtual osteotomy plans were consistently reflected in postoperative radiographic outcomes in most of the cases. The authors hypothesized that the clinical success of surgical intervention depended on the accuracy of preoperative planning and intraoperative execution. The use of patient-specific intraoperative positioning devices further improved surgical precision. An individual patient data (IPD) meta-analysis showed that 3D-guided corrective osteotomies in the treatment of DRMs lead to satisfactory radiographic and functional outcomes.^36^ Three papers directly compared preoperative virtual osteotomy plans with achieved postoperative results to assess accuracy.^12,30,32^ Kunz et al^12^ reported that their planned alignment of 26° radial inclination, 4° volar tilt, and 1 mm ulnar variance was closely matched postoperatively, with achieved values of 26° radial inclination, 4° volar tilt, and 2 mm ulnar variance. Stockmans et al demonstrated excellent precision in achieving the planned corrections for DRM with deviations in postoperative alignment from the preoperative plans of 0 ± 1 mm for ulnar variance and −1° ± 5° for radial inclination. Volar tilt proved more challenging to correct, with an average deviation of −6° ± 6°.^32^ Rieger et al^30^ demonstrated a strong correlation between virtual planning and postoperative radiographic outcomes for radial inclination and volar tilt (*r* = 0.84, *P* = .001) and a moderate correlation for ulnar variance (*r* = 0.32, *P* = .3). These findings show that 3D virtual osteotomy planning can be applied intraoperatively with a high degree of precision and may help improve clinical outcomes over less accurate techniques.

In a study outside this review, Delbrück et al also showed that postoperative alignment significantly improved, with 87.3% ± 13.8% of points falling within the ±3 mm interval compared with 48.9% ± 16.6% preoperatively (*P* = .004). Maximum and minimum deviations also decreased significantly (4.5 ± 1.1 mm and −4.5 ± 1.2 mm, respectively). In addition, a significant correlation (*P* = .004, rho = 0.928) was observed between 3D accuracy and pain reduction (ΔVAS), with higher accuracy (>90%) linked to more than 60% improvement in either DASH or Visual Analogue Scale scores for pain. These findings highlight the clinical relevance of precise 3D planning and accurate surgical execution.^37^

When discussing the accuracy and clinical impact of 3D planning in the treatment of DRM, it is important to compare with traditional methods which rely on the use of 2D planning techniques. This is beyond the scope of the current review. In addition, due to cost and time involved, 3D planning techniques have likely been reserved for more complex cases, making direct comparisons between patient groups challenging.

A recent systematic review did successfully compare 53 studies including 1003 conventional and 254 3D-guided distal radius osteotomies. In this review, the use of 3D guidance lead to higher improvements in DASH score and fewer complications compared with conventional methods.^38^ A single randomized controlled trial comparing the 2D versus 3D-guided osteotomies also found a similar result, with higher improvements of DASH scores in the 3D-guided planning group.^39^

Some added benefits of 3D-guided planning are reduced operative time, less intraoperative blood loss, and decreased intraoperative fluoroscopy use.^40^ Bauer et al^40^ found that operative time was significantly shorter in the 3D-guided osteotomy group compared with the conventional group (108 minutes [SD 26] vs 140 minutes [SD 37]; *P* < .05). Similarly, Buijze et al^39^ reported a slightly reduced operative time for 3D-guided osteotomies (91 minutes [SD 32] vs 97 minutes [SD 34]; *P* = .58), though the difference was not statistically significant. Notably, Buijze et al^39^ also observed a significantly shorter intraoperative fluoroscopy time in the 3D-guided group (58 seconds [SD 38] vs 140 seconds [SD 101]; *P* = .01). However, both studies lacked sufficient power and emphasized the need for a large trial to clearly define the clinical benefits of the 3D-guided technique.

Our systematic review found a considerable rate of complications of 11%, which aligns with rates reported in the literature for distal radius corrective osteotomies and likely reflects the overall complexity of this procedure regardless of preoperative planning method used. Commonly reported complications were extensor tendon irritation or rupture, and hardware removal with dorsal plating being more at risk compared with volar fixation.^26^ Complications reported in this review are listed in Table 2.

The use of 3D-guided preoperatively planned osteotomies has certain disadvantages that warrant consideration. These include the need for specialized software, the time and effort required for preoperative planning, the need for CT scanning of both arms, and the additional costs associated with custom-made templates and models.

With advancements in 3D printing technology, many of the techniques reviewed in this study are now commercially accessible. Companies such as Willco BV (Maastricht, The Netherlands) and Materialise NV (Leuven, Belgium) offer services to create patient-specific cutting guides using CT data and input from the treating surgeon. The prices of 3D planned services vary depending on the region and indication. However, it is estimated that it costs 2500 to 3800€ (2900-4350 USD) for 3D planning and production.^8,37^ The entire process, including virtual planning and the production of custom implants, typically takes 6 to 8 weeks, depending on the complexity of the malunion.^36^ Given the demonstrated benefit of accuracy in surgical and clinical outcomes, it is likely that future osteotomies will increasingly incorporate 3D planning techniques. One of the studies included in this review, Schindele et al, reported that the total cost for 3D-planned corrective surgery was approximately 4300 USD for DRM correction and 7000 USD for combined radius and ulna correction. This included CT imaging (323 USD for one side or 485 USD for both sides), preoperative planning, 3D printing of surgical guides, and production of custom titanium plates.^24^

Considering the additional preparation time and production costs associated with the 3D-guided workflow, further research is needed to evaluate its overall benefit. This evaluation should consider its potential benefits, including reduced operative and intraoperative fluoroscopy times, quicker return to work leading to lower productivity losses, and decreased direct medical costs due to reduced health care utilization. These factors must be carefully weighed before supporting the widespread adoption of 3D-guided techniques.

## Conclusions

Corrective osteotomies planned with 3D technology are highly accurate and demonstrate improvements in clinical outcomes for patients with DRM. With advancements and more widespread availability of 3D printing technology, this approach shows promise for treating complex malunions of the distal radius. Future research should focus on balancing the technique’s additional costs and logistical demands with its potential benefits, including improved functional outcomes, reduced complications, and cost-effectiveness in the long term.

## Supplemental Material

sj-docx-1-han-10.1177_15589447251352001 – Supplemental material for Three-Dimensional Preoperative Planning of Corrective Osteotomies for Distal Radius Malunions: A Systematic Review of Clinical and Radiographic Outcomes Supplemental material, sj-docx-1-han-10.1177_15589447251352001 for Three-Dimensional Preoperative Planning of Corrective Osteotomies for Distal Radius Malunions: A Systematic Review of Clinical and Radiographic Outcomes by Khaled Skaik, Abdulrahman Khizindar, Qasim Mughal, Marie Gdalevitch and Jennifer Mutch in HAND

sj-docx-2-han-10.1177_15589447251352001 – Supplemental material for Three-Dimensional Preoperative Planning of Corrective Osteotomies for Distal Radius Malunions: A Systematic Review of Clinical and Radiographic Outcomes Supplemental material, sj-docx-2-han-10.1177_15589447251352001 for Three-Dimensional Preoperative Planning of Corrective Osteotomies for Distal Radius Malunions: A Systematic Review of Clinical and Radiographic Outcomes by Khaled Skaik, Abdulrahman Khizindar, Qasim Mughal, Marie Gdalevitch and Jennifer Mutch in HAND

sj-docx-3-han-10.1177_15589447251352001 – Supplemental material for Three-Dimensional Preoperative Planning of Corrective Osteotomies for Distal Radius Malunions: A Systematic Review of Clinical and Radiographic Outcomes Supplemental material, sj-docx-3-han-10.1177_15589447251352001 for Three-Dimensional Preoperative Planning of Corrective Osteotomies for Distal Radius Malunions: A Systematic Review of Clinical and Radiographic Outcomes by Khaled Skaik, Abdulrahman Khizindar, Qasim Mughal, Marie Gdalevitch and Jennifer Mutch in HAND
