# Supplementary material for: Three-Dimensional Preoperative Planning of Corrective Osteotomies for Distal Radius Malunions: A Systematic Review of Clinical and Radiographic Outcomes
Source: Hand (N Y). 2025 Aug 12;21(6):1012–23. doi: 10.1177/15589447251352001 (PMC12343530; doi:10.1177/15589447251352001)
Supplement: Supplementary file 2 [file sj-docx-2-han-10.1177_15589447251352001.docx]

| Authors | Were patient’s demographic characteristics clearly described? | Was the patient’s history clearly described and presented as a timeline? | Was the current clinical condition of the patient on presentation clearly described? | Were diagnostic tests or assessment methods and the results clearly described | Was the intervention(s) or treatment procedure(s) clearly described? | Was the post-intervention clinical condition clearly described? | Were adverse events (harms) or unanticipated events identified and described? | Does the case report provide takeaway lessons? | Sum |
| --- | --- | --- | --- | --- | --- | --- | --- | --- | --- |
| Murase et al. | yes | yes | yes | yes | yes | yes | no | yes | 7 |
| Honigmann et al. | yes | yes | yes | yes | yes | yes | no | yes | 7 |
| Kunz et al. | no | yes | yes | yes | yes | yes | yes | yes | 7 |
| Oka et al. (2008) | yes | yes | yes | yes | yes | yes | no | yes | 7 |
| Temmesfeld et al. | Yes | yes | yes | yes | yes | yes | yes | yes | 8 |

**Supplemental Table S2- Case Reports**
