# Supplementary material for: Three-Dimensional Preoperative Planning of Corrective Osteotomies for Distal Radius Malunions: A Systematic Review of Clinical and Radiographic Outcomes
Source: Hand (N Y). 2025 Aug 12:15589447251352001. Online ahead of print. doi: 10.1177/15589447251352001 (PMC12343530; doi:10.1177/15589447251352001)
Supplement: sj-docx-1-han-10.1177_15589447251352001 – Supplemental material for Three-Dimensional Preoperative Planning of Corrective Osteotomies for Distal Radius Malunions: A Systematic Review of Clinical and Radiographic Outcomes [file sj-docx-1-han-10.1177_15589447251352001.docx]

| Authors | Were there clear criteria for inclusion in the case series? | Was the condition measured in a standard, reliable way for all participants included in the case series? | Were valid methods used for identification of the condition for all participants included in the case series? | Did the case series have consecutive inclusion of participants? | Did the case series have complete inclusion of participants? | Was there clear reporting of the demographics of the participants in the study? | Was there clear reporting of clinical information of the participants? | Were the outcomes or follow up results of cases clearly reported? | Was there clear reporting of the presenting site(s)/clinic(s) demographic information? | Was statistical analysis appropriate? | Sum |
| --- | --- | --- | --- | --- | --- | --- | --- | --- | --- | --- | --- |
| Haandrikman et al. | Yes | Yes | Yes | Unclear | Yes | Yes | Yes | Yes | No | Not applicable | 7 |
| Bilic et al. | Yes | Yes | Yes | Unclear | Yes | Yes | Yes | Yes | No | Yes | 8 |
| Belloti et al. | Yes | Yes | Yes | Unclear | Yes | Yes | Yes | Yes | No | Not applicable | 7 |
| Athwal et al. | Yes | Yes | Yes | Unclear | yes | yes | Yes | yes | No | Not applicable | 7 |
| Dobbe et al. | Yes | Yes | Yes | Unclear | yes | yes | yes | Yes | No | yes | 8 |
| Singh et al. | Yes | Yes | Yes | Unclear | No | Yes | Yes | Yes | No | Yes | 7 |
| Schindele et al. | Yes | Yes | Yes | unclear | No | Yes | Yes | Yes | No | yes | 7 |
| Oka et al. (2019) | Yes | Yes | Yes | yes | No | Yes | Yes | Yes | No | Yes | 8 |
| Vlachopoulos et al. | Yes | Yes | Yes | Yes | yes | Yes | Yes | Yes | No | Yes | 9 |
| Oka et al. (2018) | Yes | Yes | Yes | Yes | Yes | Yes | Yes | Yes | No | Yes | 9 |
| Yoshii et al. | Yes | Yes | Yes | Yes | Yes | Yes | Yes | Yes | No | yes | 9 |
| Athlani et al. | Yes | Yes | Yes | Yes | Yes | Yes | Yes | Yes | No | Yes | 9 |
| Miyake et al. | yes | Yes | Yes | Yes | Yes | Yes | Yes | Yes | No | Yes | 9 |
| Rieger et al. | Yes | Yes | Yes | Yes | yes | No | Yes | Yes | No | Yes | 8 |
| Schweizer et al. | Yes | Yes | Yes | Yes | Yes | Yes | Yes | Yes | Yes | Yes | 10 |
| Stockmans et al. | Yes | Yes | Yes | yes | Yes | Yes | Yes | Yes | No | Yes | 9 |
| Walenkamp et al. | Yes | Yes | Yes | Yes | Yes | Yes | Yes | Yes | Yes | Yes | 10 |
| Oka et al. (2020) | Yes | Yes | Yes | Yes | Yes | Yes | Yes | Yes | No | Yes | 9 |
| Oka et al. (2010) | yes | Yes | Yes | Yes | Yes | Yes | Yes | Yes | No | Yes | 9 |
