# Supplementary material for: Three-Dimensional Preoperative Planning of Corrective Osteotomies for Distal Radius Malunions: A Systematic Review of Clinical and Radiographic Outcomes
Source: Hand (N Y). 2025 Aug 12:15589447251352001. Online ahead of print. doi: 10.1177/15589447251352001 (PMC12343530; doi:10.1177/15589447251352001)
Supplement: sj-docx-3-han-10.1177_15589447251352001 – Supplemental material for Three-Dimensional Preoperative Planning of Corrective Osteotomies for Distal Radius Malunions: A Systematic Review of Clinical and Radiographic Outcomes [file sj-docx-3-han-10.1177_15589447251352001.docx]

**Supplemental Material:**

Search strategies:

("3D" OR "Three-dimensional" OR "3-dimensional" OR “3-D” OR “Three dimensional” OR "computer-assisted" OR "computer assisted" OR "Computer aided" OR "Patient specific instrumentation" OR "virtual planning" OR "3-D" OR "computer planning") AND (“Colles’ Fracture” OR “radius fracture”[Mesh] OR "Madelung" OR "Madelung deformity"]).

("3D" OR "Three-dimensional" OR "3-dimensional" OR “3-D” OR “Three dimensional” OR "computer-assisted" OR "computer assisted" OR "Computer aided" OR "Patient specific instrumentation" OR "virtual planning" OR "3-D" OR "computer planning") AND (Colles’ Fracture [Mesh] OR “Distal radius fracture” OR “Distal radius malunion” OR “radius fracture” OR "Madelung" OR "Madelung deformity").

("Bones of Upper Extremity"[Mesh] OR Upper limb) AND ("Osteotomy"[Mesh] OR "Corrective Osteotomy") AND ("3D" OR "Three-dimensional" OR "3-dimensional").

“Bones of Upper Extremity"[Mesh]: humerus, radius, ulna, clavicle, scapula (with the acromion, coracoid process, and glenoid cavity), carpal bones, metacarpal bones, and finger phalanges.

“Osteotomy"[Mesh]: orthognathic surgical procedures, alveolar bone grafting, Le Fort osteotomy, and sagittal split ramus osteotomy.

“Radius Fracture”[Mesh]: Radial Head and Neck Fractures.
